# Retinal Cell Degeneration in Animal Models

**DOI:** 10.3390/ijms17010110

**Published:** 2016-01-15

**Authors:** Masayuki Niwa, Hitomi Aoki, Akihiro Hirata, Hiroyuki Tomita, Paul G. Green, Akira Hara

**Affiliations:** 1 Medical Science Division, United Graduate School of Drug Discovery and Medical Information Sciences, Gifu 501-1194, Japan; 2 Department of Tissue and Organ Development, Gifu University Graduate School of Medicine, Gifu 501-1194, Japan; hito7ao@gifu-u.ac.jp; 3 Division of Animal Experiment, Life Science Research Center, Gifu University, Gifu 501-1194, Japan; akatsuki@gifu-u.ac.jp; 4 Department of Tumor Pathology, Gifu University Graduate School of Medicine, Gifu 501-1194, Japan; h_tomita@gifu-u.ac.jp (H.T.); ahara@gifu-u.ac.jp (A.H.); 5 Department of Oral and Maxillofacial Surgery, University of California at San Francisco, San Francisco, CA 94143, USA; Paul.Green@ucsf.edu

**Keywords:** retinal cell degeneration, NMDA, CoCl_2_, EAE, ischemia, nerve crush, light-induced, animal model

## Abstract

The aim of this review is to provide an overview of various retinal cell degeneration models in animal induced by chemicals (*N*-methyl-d-aspartate- and CoCl_2_-induced), autoimmune (experimental autoimmune encephalomyelitis), mechanical stress (optic nerve crush-induced, light-induced) and ischemia (transient retinal ischemia-induced). The target regions, pathology and proposed mechanism of each model are described in a comparative fashion. Animal models of retinal cell degeneration provide insight into the underlying mechanisms of the disease, and will facilitate the development of novel effective therapeutic drugs to treat retinal cell damage.

## 1. Introduction

Diseases that cause visual dysfunction may be divided into functional (e.g., ametropia, myopia, hyperopia, astigmatism, *etc.*), dysregulation functional (e.g., presbyopia) and organic (e.g., cataracts and glaucoma) categories, with retinal disease being the most common cause of blindness. In particular, glaucoma is an example of a disease that involves retinal neuronal death, and it has been believed that the underlying pathology is due to increased intraocular pressure followed by obstruction of aqueous humor outflow. In addition to this mechanical cause of glaucoma pathology, glutamate neurotoxicity is also thought to be an important cause of retinopathy in glaucoma [1,2].

In many eye diseases that lead to blindness, retinal nerve cell degeneration is a major factor as a direct cause of vision loss. Ultimately, it is necessary to identify the basic pathology of these retinal diseases in order to develop novel treatments. However, clinical data alone does not provide a complete understanding of the pathological processes underlying retinal diseases, therefore use of appropriate preclinical models has been used to provide a better understanding of the underlying pathologies. The results of many lines of basic research have indicated that ischemia, glutamic acid disorders, inflammation, nutritional factor deficiency, oxidative stress, *etc.* may be involved in the nerve cell degeneration that occurs in retinal disease.

Glaucoma is a disease that results in mechanical depression of the optic nerve head, leading to tunnel vision, and, if left untreated, progressive retinal neuropathy, visual impairment and, eventually, blindness. Glaucoma has been often associated with elevated intraocular pressure (IOP), producing ischemia, which results in glutamate release, and this initiates the death of neurons that express *N*-methyl-d-aspartate (NMDA)-type glutamate receptors (e.g., ganglion cells and a subset of amacrine cells [3]). However, recent evidence suggested that there were no significant differences in vitreal glutamate concentration between normal control eyes and glaucomatous eyes both in monkeys and in human [4,5]. Furtheremore, IOP elevation is not detected in a significant subset of individuals with glaucoma, such as those classified with normal tension glaucoma (NTG). NTG is a subset of primary open-angle glaucoma that exhibits statistically normal intraocular pressure, but shows glaucomatous optic neuropathy and results in visual field defects. Epidemiological studies indicate that NTG represents 20%–90% of all primary open-angle glaucoma, with percentages varying by race [6,7]. Interestingly, IOP still seems to play a role in normal tension glaucoma because a substantial number of patients with NTG as well as other forms of primary open-angle glaucoma benefit from lowering of intraocular pressure [8]. Thus, NTG may be caused by the vulnerability of optic nerves to normal range of intraocular pressure. In addition, in another subset of individuals with glaucoma, reducing IOP does not prevent disease progression. Thus, it is important to understand the pathophysiology of IOP-independent mechanisms that lead to retinal ganglion cell (RGC) loss. Many factors such as glutamate excitotoxicity, increased matrix metalloproteinase expression, TNF-α upregulation, increased nitric oxide synthase-2 expression, and oxidative stress are largely included in the molecular mechanism of glaucoma, although the pathopyisiology of glutamate optic neuropathy is not well understood in the present time [9].

## 2. NMDA-Induced Retinal Ganglion Cell Degeneration

Glutamate is an excitatory neurotransmitter, and excessive extracellular glutamate in glaucoma stimulates NMDA receptors (NMDAR), which are involved in the retinal neuronal cell death [10,11,12]. Loss of RGCs is a distinctive feature of several retinal diseases, such as glaucoma, retinal ischemia, and diabetic retinopathy [13,14,15]. Since RGCs have high permeability to calcium ions, it is believed that NMDAR-mediated excitotoxicity plays a major role in RGC death [16].

NMDARs form heterotetramers of two GluN1 and two GluN2 subunits, with GluN2 subunits (GluN2A–D) being the major determinants of the pharmacological and biophysical properties of NMDARs. All NMDAR subunits are expressed in RGCs in the retina. In glutamate aspartate transporter-deficient mice, NMDA-induced excitotoxic retinal cell death and RGC degeneration is primarily mediated by GluN2B- and GluN2D- but not GluN2A- or GluN2C-containing NMDARs [12]. Thus, inhibiting GluN2B and GluN2D activity may be a novel therapeutic approach for treating certain retinal diseases.

The glutamate transporter is the only mechanism by which glutamate is removed from the extracellular fluid in the retina [17]. Harada *et al.* reported that mice deficient in the glutamate transporters glutamate/aspartate transporter (GLAST) or excitatory amino acid carrier 1 (EAAC1) demonstrate spontaneous RGC loss and optic nerve degeneration without elevated IOP, while administration of glutamate receptor blocker prevented RGC loss [18].

During development and following axonal damage, exposure to excitotoxins and other pathological situations, RGCs and other retinal neurons die by apoptosis. It is believed that glutamate-induced excitotoxicity is responsible for the selective loss of retinal neurons after retinal ischemia as well as in glaucoma [19]. Intravitreal injection of NMDA is a good model for *in vivo* neuronal apoptosis [10,20,21]. NMDA causes fragmentation of internucleosomal retinal neuron DNA as well as apoptosis-specific activation of the enzyme, caspase-3 [21,22]. Interestingly, we observed fragmented DNA transport in dendrites of retinal neurons during apoptotic cell death induced by intravitreal NMDA injection [23]. It has been hypothesized that similar DNA transport may occur in other forms of neuronal apoptosis, since a similar phenomenon occurs in dendrites of gerbil hippocampal CA1 pyramidal neurons during ischemia-induced apoptosis [24]. Therefore, it is possible that the movement of fragmented DNA affects regulation and maintenance of retinal neuronal networks. Interestingly, endogenous tPA, but not uPA, acts as a facilitator in NMDA-induced RGC cell loss, but the mechanism of this does appear to be associated with cleavage of plasminogen into plasmin in the fibrinolytic cascade [25,26,27].

Thus, animal models of NMDA-induced retinal cell degeneration, such as intraocular NMDA injection, glutamate transporter or specific NMDAR deficit mice, are useful models of RGC loss not only for drug discovery research [28,29,30,31,32,33,34,35], but for research into the regeneration of RGC [36,37]. For example, we have attempted retinal regeneration by transplantation of human embryonic stem cells after RGC loss induced by NMDA injection [38,39]. Differentiated embryonic stem (ES) cells growing along the retinal surface 30 days after transplantation developed fine neuronal cell processes around cell nuclei and neuronal networks extended into the retinal inner plexiform layer [40]. We have also reported *in vivo* differentiation of human ES cells into retinal ganglion-like cells in NMDA-induced RGC mouse model [41,42]. ES cells may be useful for neural tissue regeneration in the adult mammalian retina although it will be necessary to control teratoma growth. To reduce the teratoma formation and to induce neuronal differentiation after ES cells implantation in adult mice [38] and in nude mice [39], the folate antagonist methotrexate appears to be a very useful tool for cell-replacement therapy.

## 3. CoCl_2_-Induced Retinal Photoreceptor Cell Degeneration

The majority of genetic mutations that result in retinal degeneration affect both the retinal pigment epithelium as well as sensory retina. For example, retinitis pigmentosa, which is a cause of blindness and visual impairment in younger people, is characterized by a gradual loss of photoreceptors through incompletely understood mechanisms [43]. Mutations in a number of different genes (including rhodopsin, the beta subunit of cGMP phosphodiesterase and peripherin) have been identified as the primary genetic lesion in different forms of retinitis pigmentosa [44]. Age-related macular degeneration (AMD) is a complex genetic disorder that involves the retinal pigment epithelium and mutations in one or more genes that contribute to an individual's susceptibility for developing the disease [45,46]. To date, there are no cures for these genetic diseases, and successful future treatments based on cell replacement or gene therapy will only be achieved if we have a greater understanding of the underlying pathophysiological processes [47,48]. To this end, animal models of retinal degeneration have been developed, and use of these models has led to a better understanding of disease pathology and to the development of possible therapeutic strategies. A well-established animal model of retinal degeneration, the rd mouse model, involves a mutation of the rod-specific phosphodiesterase that leads to the rapid and marked death of rod photoreceptors within the first few postnatal weeks [49]. Within 2 months, this loss of rod photorecptors results in subsequent cone degeneration and blindness [50]. Other animal models of spontaneous retinal degeneration have been discovered by screening mice from genetically independent mouse strains [51,52]. While the role of genes in retinal degeneration is well established [43,53,54], less is known about environmental and metabolic factors that contribute to the degenerative process. The loss of photoreceptors themselves produce metabolic changes in the remaining retina, and it has been hypothesized that the local retinal oxygen environment is an underlying pathophysiological factor [48], since manipulation of environmental oxygen levels modulates the rate of photoreceptor degeneration [53,54].

**Figure 1:**
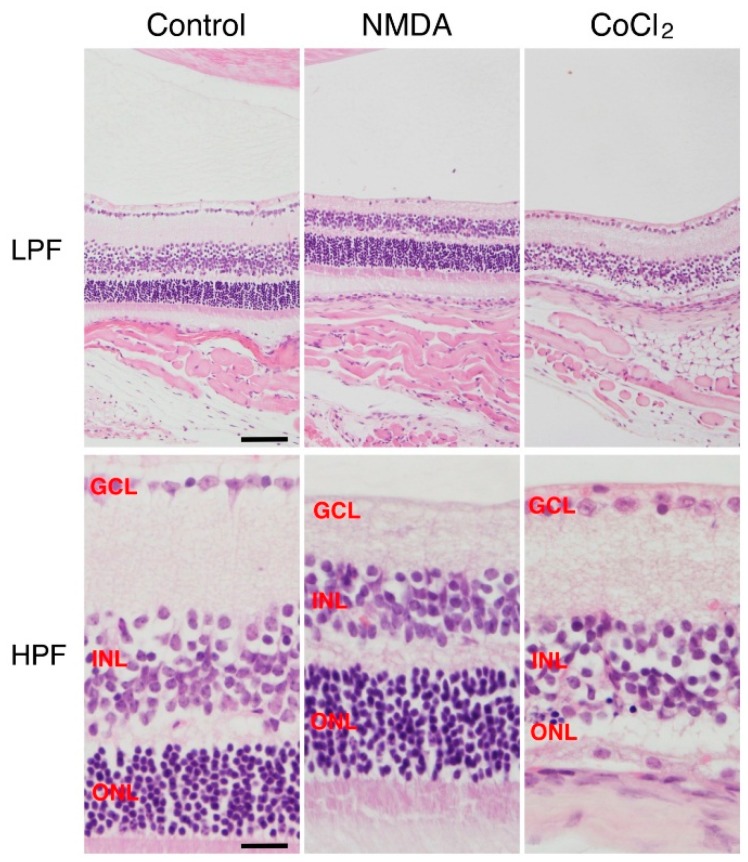
Retinal cell degeneration induced by NMDA and CoCl_2_ in mice. Representative photographs show a vehicle-treated normal control retina, NMDA-treated retina (15 mM NMDA, 2 μL/eye) and CoCl_2_-treated retina (9 mM CoCl_2_, 2 μL/eye) at 7 days after intravitreal injection. The NMDA-treated retina demonstrates disappearance of ganglion cells in GCL and CoCl_2_-treated retina shows degeneration of photoreceptor cells in ONL. LPF, low-power field; HPF, high-power field; NMDA, N-methyl-d-aspartate; GCL, ganglion cell layer; INL, inner nuclear layer; ONL, outer nuclear layer. Scale bars in left upper and left lower panels indicate 80 μm and 20 μm, respectively.

Cobalt chloride (CoCl_2_) has been widely used as a hypoxia-mimicking agent in both *in vivo* [55] and *in vitro* studies [56]. Both cobalt and hypoxia affect a similar group of genes on a global gene expression level [57,58,59]. Cobalt is essential for human health because of its critical role in the synthesis of vitamin B12 [60]; however, excess exposure of cobalt can lead to tissue and cellular toxicity. CoCl_2_ binds directly to HIF-1α thereby inhibiting its binding to the von Hippel-Lindau protein, (a mediator of HIF-1α degradation) and and causing HIF-1α to accumulate. In addtion, CoCl_2_ gives rise to local hypoxia [61]. Hypoxia produced by chemical agents is a widely used model [62,63,64], since it is easy to control the level of hypoxia by varying the concentration of the hypoxic agent, but, at least in studies involving the retina, CoCl_2_ has mainly been used *in vitro* experiments. We have used a low dose of CoCl_2_ in *in vivo* studies, performing intravitreal injection of CoCl_2_ to induce selective photoreceptor cell degeneration in mice (Figure 1) and rats, which produces DNA fragmentation in the outer nuclear layer [65]. Interestingly, Hara *et al.* reported that expression of septins 8, one of cytoskeletal GTP-binding proteins restricted to the nuclei of photoreceptor cells of mice and rats, was decreased after intravitreal injection of CoCl_2_, and its disappearance was concomitant with photoreceptor cell degeneration [66]. This implies that septin 8 has specific key functions in retinal photoreceptor cells.

The retinal morphology observed 2 weeks after CoCl_2_ injection mimics retinal degeneration seen in the mutant rd mouse model, suggesting that photoreceptor cell degenerations in both the genetic and metabolic models at least some of the same pathophysiological processes. In addition to manipulating the oxygen environment to affect photoreceptor cells degeneration, CoCl_2_-treated animals can be used to study tissue regeneration. The rd mouse is commonly used as a recipient animal to assess the capacity of grafted neural progenitor cells [67] or embryonic stem cells [68] to grow. However, as an alternative to using animals with gene mutations, the CoCl_2_ injection model may be also used to evaluate tissue regeneration.

Of note, by using a green fluorescent vascular endothelium zebrafish transgenic line treated with CoCl_2_ 24 h postfertilization, Wu and colleages described a potential retinopathy of prematurity model; they observed, a significantly increase in the number of vascular branches and sprouts in the central retinal vascular trunks [69]. A clearer understanding of the mechanism of prematurity retinopathy development may come from use of this simple zebrafish model of prematurity retinopathy might provide, and may facilitate research into new treatment methods.

## 4. Demyelination and Retinal Cell Degeneration of Experimental Autoimmune Encephalomyelitis (EAE) as a Model for Multiple Sclerosis

Multiple sclerosis (MS) is an immune-mediated, demyelinating and neurodegenerative disease that currently lacks any neuroprotective therapies. Since the visual system is a relatively accessible sensory system that includes white matter that is affected by neuroinflammatory process in MS, it provides a good target to monitor nerve loss and repair. Optic neuritis, which occurs as a result of inflammation of the optic nerve and is often the presenting feature of MS, can result in loss of vision due to impulse conduction failure through demyelination [70,71], axonal transection and loss in the optic nerve followed by depletion of retinal ganglion cells (RGC) in the retina. Optic neuritis is associated with retinal nerve fibre layer thinning during the progression of MS, and individuals with MS develop retinal nerve loss with regard to the diagnosis of optic neuritis [72]. Therefore, monitoring axonal loss due to optic neuritis would be a valuable method of assessing the effectiveness of therapeutic strategies targeting neurodegeneration during inflammatory central nervous system (CNS) diseases [73].

Experimental autoimmune encephalomyelitis (EAE) is the most commonly used paradigm for MS, and can be induced in mice by immunization with myelin antigens [74]. Neuronal loss, including RGC apoptosis in eyes with optic neuritis, also occurs in EAE [75]. However, the clinical course and level of neuronal damage in MS and EAE is variable. A study of mechanisms and kinetics of RGC loss in C57/BL6 mice immunized with myelin oligodendrocyte glycoprotein to induce a chronic EAE disease indicated that differences in the course of clinical disease during optic nerve inflammation may trigger distinct mechanisms of neuronal damage, or RGCs in different rodent strains may have variable resistance to neuronal degeneration [76].

Infiltration of inflammatory cells mediate demyelination and axonal injury in EAE models of experimental optic neuritis, and axonal injury triggers RGC death occurs by apoptosis. EAE not only affects activation of apoptotic signals, but also causes a glial response in the retina [77]. Neuroprotective therapies will likely need to be initiated prior to axonal injury to prevent permanent RGC loss from optic neuritis to preserve neuronal function [78]. There are many reports that have described prevention of RGC loss in EAE. For example, corticosteroids attenuated both optic neuritis and RGC loss when treatment was initiated before onset of optic nerve inflammation, EAE. Once inflammation has started, corticosteroids are less effective, suggesting that chronic immunomodulation may prevent recurrent optic neuritis and RGC damage [79]. HE3286, (17α-ethynyl-5-androstene-3β,7β,17β-triol), a synthetic derivative of a natural steroid, exerts anti-inflammatory effects in several disease models, suppresses inflammation, reduces demyelination and axonal loss, and promotes RGC survival and preservation of function [80]. Calpain, a member of calcium-dependent, non-lysosomal cysteine protease family that is ubiquitously expressed in mammals, inhibits expression of proapoptotic proteins and the proinflammatory molecule, nuclear NF-κB, in the retina of Lewis rats with acute EAE. These data indicate that calpain inhibition might be a useful supplement to immunomodulatory therapies such as corticosteroids in optic neuritis, due to its neuroprotective effect on RGCs [81,82]. Brambilla *et al.* reported transgenic inhibition of astroglial NF-κB ameliorates optic nerve damage and also retinal ganglion cell loss in experimental optic neuritis [83] suggesting that the NF-κB system may play a crucial role in EAE development. Administration of NMDA receptor antagonists protects RGC and axons, as well as reduces optic nerve demyelination in EAE, indicating that NMDA receptor blockade protected RGCs directly and that the protection is independent of effects on oligodendrocytes. Moreover, increased RGC survival was observed before the onset of optic nerve demyelination—when RGC degeneration had already started. These results indicate an important pathophysiological role for NMDA receptor-mediated glutamate toxicity during the induction phase of this disease model and highlight a potential target for therapeutic neuroprotection in human optic neuritis [84]. Furthermore, it has also been reported that inflammatory damage of axons in the optic nerve and subsequent loss of RGCs in the retina in EAE was inhibited by the systemic administration of a sodium channel blocker (oxcarbazepine) or intraocular treatment with siRNA targeting caspase-2 [74] and by an N-type voltage-dependent calcium channel blocker [85]. Importantly, recent evidence suggested that the most widely used disease-modifying drugs for MS targeting inflammation are ineffective in preventing permanent disability [86]. Interestingly, Talla *et al.* has reported that NADH-dehydrogenase type-2 expression in mitochondria of RGCs suppressed irreversible visual loss and retinal cell degeneration in the EAE animal model of MS [87]. Since mitochondrial dysfunction has been proposed as a cause of axonal degeneration in MS patients [88], targeting the dysfunctional NADH-dehydrogenase type-2 of EAE responsible for loss of respiration, mitochondrial oxidative stress and apoptosis may be a novel approach to ameliorate neuronal and axonal loss that that is not addressed by current disease modifying drugs for MS [87].

Besides the EAE model, toxin-induced demyelinating models, such as the cuprizone (bis-cyclohexanone-oxalyldihydrazone) [89,90], lysophosphatidyl choline [91] and the ethidium bromide [92] models, have been used to study the molecular factors contributing to demyelination processes. The cuprizone model has been used as a retinal demyelination model, and Namekawa *et al.* [93] reported that cuprizone demyelinates optic nerves and the extent of demyelination is attenuated in mice overexpressing Dock3, an atypical guanine nucleotide exchange factor. Moreover, impairment of visual function by cuprizone treatment (as demonstrated by multifocal electroretinograms) is prevented by Dock3 overexpression.

## 5. Retinal Cell Degeneration Induced by Optic Nerve Crush Injury

Injury to the optic nerve (ON) is associated with various ocular diseases and abnormalities, such as glaucoma, traumatic optic neuropathy, ischemic optic neuropathy, and compressive optic neuropathy, all of which lead to RGC loss via apoptosis [94]. One animal model in widespread use for the study of RGC loss is optic nerve crush, which leads to the initiation of caspase cascades and apoptosis of RGC in a predictable manner [95]. The involvement of specific caspases in optic neuropathy, glaucoma, and RGC death has been previously implicated [96]. For example, in the ON of glaucoma patients, more axons were found to express caspase-3, while in the animal model, ON crush activates caspase-2 [97] and ON axotomy activates caspases-3, -6, -8, and -9 in RGCs [98,99,100,101,102]. More recently, Choudhury *et al.* reported caspase-7 is a critical mediator of optic nerve crush-induced RGC death [96]. Additionally, in another animal models, it has been reported that chronic ocular hypertension in rat model activates caspases-3, -8, and -9 [98,99,100,101,102,103,104,105] and retinal transient ischemia activates caspases-2 and -3 [106,107]. Various compounds were studied for development research using this optic nerve crush model [108,109,110,111]. For example, Dock3 overexpression and p38 inhibition synergistically stimulate neuroprotection and axon generation after optic nerve crush [112].

It has been reported that trophic factors, such as brain-derived neurotrophic factor (BDNF), protect RGCs and promote axon regeneration in an ON model [113]. Recently, Harada *et al.* [114] reported that glial BDNF signaling plays an important role in retinal glial cells in the early stage of neural protection after ON injury using TrkB receptor deleted mice. A similar result was also reported in glutamate-induced retinal degeneration model [115].

In fish, persistent neurogenesis and the capacity to regenerate neurons leading to repair and restoration of function in a damaged retinal tissue of adult animals has been recognized for decades. Most recent studies of persistent neurogenesis and regeneration in the retina have used the zebrafish as an experimental model because of the many well-known advantages of this species: ease of maintaining and breeding, the availability of genetic tools including mutants and transgenic lines, and a rapidly growing community of zebrafish researchers [116]. Typically, nerve injury of adult mammalian CNS neurons results in retrograde neuronal degeneration and cell death. However, while the RGCs of rats do not regenerate and become apoptotic after optic nerve injury, goldfish RGCs can survive and regrow their axons after injury. Koriyama *et al.* also reported different pAkt-Bax expression in goldfish and rats after optic nerve crush as a key factor [117].

## 6. Transient Ischemia-Induced Retinal Cell Degeneration

The retina is part of the CNS, and therefore cell death pathways that are produced in response to ischemic damage in the retina mirror those found in other areas of the CNS undergoing similar trauma [118]. Among the experimental models of cerebral ischemia, the most common are focal models that either permanently or transiently occlude blood flow of middle cerebral artery (MCA) [119,120]. Filamentous MCA occlusion (fMCAO) is the most frequently used focal cerebral ischemia model in rodents [121]. Steele *et al.* reported that fMCAO induced retinal ischemic damage because the ophthalmic artery originated from the internal carotid artery and is proximal to the origin of the MCA [122]. As the ophthalmic artery predominantly supplies the inner retina and it is proximal to the origin of MCA, occlusion of the MCA will simultaneously interrupt the vascular supply to the retina and the whole eye, resulting in retinal ischemia [123]. fMCAO is a useful model for studies aimed at understanding the changes that lead to retinal damage in these patients and may be used to develop novel therapeutic agents [122].

Addition to fMCAO, many studies have shown that retinal cell damage may be induced by transient retinal ischemia, for example by increasing IOP above the arterial blood pressure [123,124,125] or by ligating the optic nerve together with the central retinal artery for some period [124,125]. These retinal transient ischemic models have been widely used for development of therapeutic compound or tools [126,127,128,129,130]. However, increasing IOP may also produce mechanical damage to neuronal cells due to the high pressure itself, whereas nerve ligation may cause indirect effects due to interruption of neuronal transport, and also to a possible ligation of collateral blood supplies. We have developed a highly reproducible transient ischemia of the rat retina produced by photochemical induction of a thrombotic occlusion, with a combination of intravenous injection of photo-sensitive Rose Bengal dye and green laser irradiation of the central retinal artery, and its subsequent thrombolytic reperfusion with tPA [131]. After the transient retinal ischemia-induced by this method, RGC loss were clearly observed [131]. The photochemically induced retinal ischemic model might be a useful tool for pharmacotherapy research.

Retinal vein occlusion (RVO) is the second most common retinal vascular disease after diabetic retinopathy [132]. However, since there is currently no definitive treatment for RVO, a reliable animal model of RVO is needed in pharmacotherapy research. For this purpose, several methods, namely mechanical ligation [133], endothelin-1 injection [134] and light coagulation [135], were used for inducing RVO in rats, cats, or rabbits. Currently, the most common method to induce RVO in rats is laser photocoagulation with a photosensitizer [136]. Recently, Chen *et al.* [137] have developed a reproducible and reliable animal rat RVO model that is produced by photochemically-induced ischemia using erythrosin B, and mimicks key features of human RVO. A similar model in miniature pig has also been developed [138].

Retinal ischemia results in irreversible morphological and functional changes, and the tissue damage and functional deficits that follow periods of transient ischemia reflect the combined effects of several interrelated pathophysiological pathways that result in drastic changes in ion movements, neurotransmitter levels and metabolites. Among them, a great deal of evidence suggested that glutamate release and activation of NMDA and non-NMDA receptors clearly play an important role in retinal ischemic injury [139]. The glutamate toxicity after retinal ischemia was caused in conjunction with other pathological cascade, such as TNF/TNF receptor 1 activation [140].

## 7. Light-Induced Retinal Cell Degeneration

Age-related macular degeneration (AMD), a common and painless eye condition, is a leading cause of vision loss for people older than 50 years, but the molecular mechanism underlying AMD is unkown [141]. Light-induced retinal damage, a well-established *in vivo* model for retinal degeneration, mimicks most of the essential characteristics of human AMD and has been widely used to investigate the mechanisms of neuroretinal dysfunction [142]. Light is a double-edged sword in the visual system; that is, light triggers the well-known visual transduction cascade by its action on rhodopsin, but light exposure can also induce apoptosis in the retinal cells including photoreceptors and RGCs [142,143]. Although the exact molecular and pathophysiologic mechanism of light-induced photoreceptor and RGC damage remain unclear, recently, several mechanisms were proposed, such as up-regulation of pyruvate kinase isozyme type M2 (PKM2) for RGC loss [144] and increased expression of the proton-sensing G protein-coupled receptor Gpr65 for photoreceptor degeneration [145]. It has also been reported that functional autophagy plays key roles of light-induced retinopathy [146].

Interestingly, it has been reported that various stressors, such as mechanical injury, bright light, and ischemia, protect the retina against light-induced photoreceptor degeneration [147,148,149]. Casson *et al.* demonstrated that RGC loss induced by optic nerve transection or intravitreal NMDA-injection protect light-induced photoreceptor degeneration [150].

## 8. Conclusions

In this review, we described various animal models of retinal cell degeneration induced by chemical, autoimmune, mechanical stress and ischemia. Their target region, pathology and proposed mechanism of each model are summarized in Table 1. Both NMDA and CoCl_2_ are used to specifically induce loss of RGCs and retinal photoreceptor cells, respectively. These chemical models are easily applied to mice and other animals. The autoimmune model, EAE, secondarily induces specific RGC loss due to demyelination and axonal injury. Optic nerve crush injury, one method of mechanical stress, induces selective RGC loss via caspase activation. Various compounds have been studied for drug development research using this model. *In vivo* retina ischemia models, which produce degeneration of RGC, photoreceptor cell and amacrine cells, are useful to study various cell death pathways such as apoptosis. Light-induced retinal degeneration mainly affects RGC and photoreceptor cells, mimics most of the essential characteristics of human AMD and has been widely used to study the mechanism of retinal dysfunction. Thus, a variety of animal models of retinal degeneration have been used to elucidate key mechanisms underlying retinal cell degeneration in order to develop novel effective therapeutic drugs to treat retinal cell damage.

**Table 1:** Summary of animal models for retinal cell degeneration.

	Target Region	Pathology	Proposed Mechanism	Reference
NMDA-induced retinal cell degeneration	RGC	RGC loss	NMDAR, (GlunN2B and Glun2D) activation glutamate transporter deficit	[10,11,12,16,18]
CoCl2-induced retinal cell degeneration	Photoreceptor	photoreceptor cell loss	HIF-1alpha, hypoxia, septin8?	[59,61,66]
Experimental autoimmune encephalomyelitis (EAE)	Myeline	demyelination and axonal injurycaused RGC loss	dysfunctional NADH-dehydrogenase type-2, Dock3	[87,88,93]
Optic nerve crush injury	Optic nerve	RGC loss	caspase activation	[95,96]
Transient ischemia-induced retinalcell degeneration	Retina	Degeneration of RGC, Photoreceptor cells andAmacrine cells	glutamate release and NMDA/non-NMDA receptor activation, Activation of TNF/TNF-R system, etc.	[139,140]
Light-induced retinal cell degeneration	RGC, photoreceptor	RGC loss photoreceptor cell loss	up-regulation of PKM2 for RGC loss increased expression of Gpr65 for photoreceptor loss	[144,145]

Retinal and optic nerve degenerative diseases are major causes of blindness. Basic preclinical research examining nerve protection and regeneration therapy in eye diseases, including glaucoma, have explored many approaches, such as use of stem cells and ES cells for transplantation in MCAO stroke model were performed [125]. We also attempted retinal regeneration by transplantation of ES cells after retinal degeneration induced by NMDA [39,40]. Overexpression of Dock3 also contributes to both neuroprotection and axon regeneration in retinal degeneration induced by optic nerve injury model and demyelinating model [95,114]. The animal models of retinal diseases described in this review have already yielded important findings, and will ultimately lead to effective novel therapies for the treatment of retinal cell degeneration.
